# An innovative cardiac rehabilitation based on the power–force–velocity profile to further improve cardiorespiratory capacities in coronary artery disease patients: CITIUS study

**DOI:** 10.1093/ehjopen/oeaf036

**Published:** 2025-04-22

**Authors:** Marie Fanget, Pierre Labeix, Jean-Benoit Morin, Manon Bayle, Jerome Koral, Rodolphe Testa, Nicolas Peyrot, Vincent Gremeaux, Marie-Christine Iliou, Thierry Busso, Jari Antero Laukkanen, Frederic Roche, David Hupin

**Affiliations:** Department of Clinical and Exercise Physiology, University of Jean Monnet, University Hospital of Saint-Etienne, Mines Saint-Etienne, INSERM, U 1059, Saint-Etienne, Saint-Priest-en-Jarez 42270, France; Department of Clinical and Exercise Physiology, University of Jean Monnet, University Hospital of Saint-Etienne, Mines Saint-Etienne, INSERM, U 1059, Saint-Etienne, Saint-Priest-en-Jarez 42270, France; Inter-university Laboratory of Human Movement Biology, EA 7424, UJM-Saint-Etienne, Saint-Priest-en-Jarez 42270, France; Department of Clinical and Exercise Physiology, University of Jean Monnet, University Hospital of Saint-Etienne, Mines Saint-Etienne, INSERM, U 1059, Saint-Etienne, Saint-Priest-en-Jarez 42270, France; Institut National du Sport, de l'Expertise et de la Performance (INSEP), Paris 75012, France; Clinique Universitaire du Sport et de l'Arthrose, Centre Hospitalier Universitaire de Saint-Etienne, Saint-Etienne 42270, France; Movement–Interactions–Performance, MIP, EA 4334, Le Mans Université, Le Mans 72085, France; Department of Sports Medicine, Swiss Olympic Medical Center, Lausanne University Hospital, Lausanne 1011, Switzerland; Institute of Sports Sciences, University of Lausanne, Lausanne 1015, Switzerland; Cardiac Rehabilitation Department, Saint-Joseph Hospital, Paris 75014, France; Inter-university Laboratory of Human Movement Biology, EA 7424, UJM-Saint-Etienne, Saint-Priest-en-Jarez 42270, France; Institute of Clinical Medicine, University of Eastern Finland, Kuopio FI-70210, Finland; Department of Internal Medicine, Wellbeing Services County of Central Finland, Jyväskylä 40620, Finland; Department of Clinical and Exercise Physiology, University of Jean Monnet, University Hospital of Saint-Etienne, Mines Saint-Etienne, INSERM, U 1059, Saint-Etienne, Saint-Priest-en-Jarez 42270, France; Department of Clinical and Exercise Physiology, University of Jean Monnet, University Hospital of Saint-Etienne, Mines Saint-Etienne, INSERM, U 1059, Saint-Etienne, Saint-Priest-en-Jarez 42270, France

**Keywords:** Cardiac rehabilitation, Physical activity, Coronary artery disease, Power–force–velocity relationship, Cycle sprint, Exercise

## Abstract

**Aims:**

Individually optimizing the training programme of cardiac rehabilitation (CR) remains a major concern among coronary artery disease (CAD) patients. The power–force–velocity profile (PFVP) for a given task is usually assessed to improve performance in athletes through individualized training. Therefore, assessing PFVP on stationary cycle ergometer may allow better personalization of CR programme. The aim of this study was to compare the effects of a new CR customized based on patient’s PFVP vs. a traditional CR in CAD patients on cardiorespiratory, biological, and muscular systems.

**Methods and results:**

A total of 86 patients participated in this study. The 3-week intervention consisted of physical training sessions (4/week) and therapeutic education workshops (1/week). Experimental group patients followed a specific cycle ergometer training programme focusing on their less developed PFVP quality. Control patients attended a conventional CR programme. Cardiopulmonary exercise test (VO_2_ at the first ventilatory threshold, VT_1_, and the peak), blood tests [LDL and HDL cholesterol (LDL-C and HDL-C)], and handgrip and quadriceps force were assessed at baseline and after CR. The mean age was 60.8 ± 9.6 years, and 15% were women. A significantly greater benefit in VO_2_  _peak_ (experimental: +21.5 ± 19.2% vs. control: +10.5 ± 15.8%, *P* < 0.001), VO_2_ at VT_1_ (experimental: +35.5 ± 33.6% vs. control: +8.4 ± 31.2%, *P* < 0.001), and LDL-C (*P* = 0.001) were observed in the experimental group. Both groups significantly increased HDL-C and muscle parameters.

**Conclusion:**

The novel CR, based on initial individual PFVP performed on stationary cycle ergometer, showed greater benefits on cardiorespiratory capacities and lipid profile than a conventional, non-individualized CR. Therefore, PFVP could be used in CR to adapt specifically the content of training sessions.

## Graphical Abstract

**Graphical Abstract:**
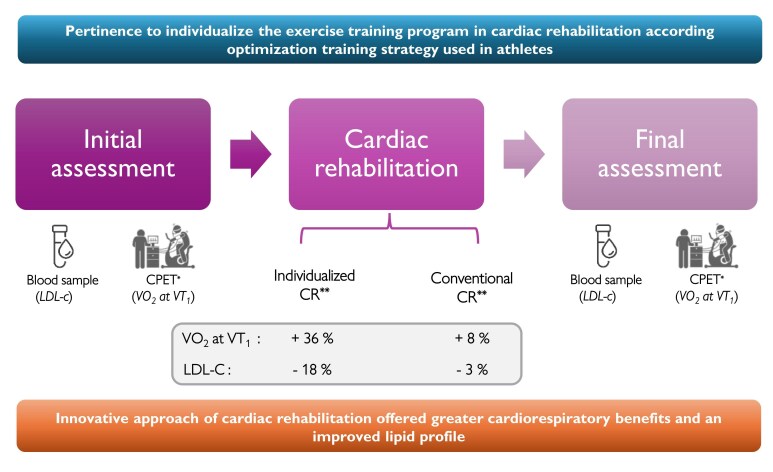

## Introduction

Secondary prevention is of utmost importance to limit the recurrence of cardiac events and reduce cardiovascular morbidity and mortality.^1^ After an acute coronary syndrome, a cardiac rehabilitation (CR) programme is recommended to maintain or increase physical capacities and decrease cardiovascular risk.^2,3^ This programme, based on a comprehensive multidisciplinary intervention, combines lifestyle education workshops, psychological assistance, and physical training.^4,5^ The latter represents the predominant part of CR.^6,7^

Optimizing the exercise training programme is constantly sought in CR. Indeed, CR has evolved over the last decades.^3,6,8^ Moderate-intensity continuous training (MICT) was first considered as the gold standard.^9,10^ In the 2000s, some studies compared physiological effects between MICT and high-intensity interval training (HIIT) among patients with cardiac disease and suggested that HIIT provided similar or superior cardiovascular adaptations than MICT.^11–13^ Indeed, continuous and interval aerobic training improves exercise duration, maximum cardiorespiratory capacity (VO_2 max_), and at the first ventilatory threshold (VT_1_).^12,14,15^ Improvement of the VT_1_ is of major clinical relevance, as it reflects the functional capacity required for daily activities and the perception of quality of life. Aerobic training also contributes to reduce anxiety and depression symptoms and improves metabolic factors (blood lipid levels).^16–19^ The latest guidelines of the European Society of Cardiology recommend a moderate- or moderate–high-intensity endurance training combined with resistance training for stable cardiac patients.^3,6^ Resistance training provides additional adaptations to aerobic training that are also beneficial to the patient. This type of training is associated with an increase in muscular strength and an improvement in body composition and metabolic syndrome.^7,20,21^

Exercise training in CR has evolved thanks to the contribution of the sport sciences. It may be thus relevant to explore strategies derived from the athletic field, such as the power–force–velocity profile (PFVP), to enhance the positive effects. This approach may notably be used by athletes to improve their performance through a more individualized athletic training.^22,23^ Specifically, the PFVP characterizes the relative part of force and velocity outputs in the production of external power for a given motor task. Hence, this assessment can highlight a potential imbalance between these two independent qualities (i.e. force or velocity deficiency). In the many physical activities, an optimal balance between these both physical qualities is required. The work may thus focus on the weaker point, while trying not to alter the main quality.^24^

To our knowledge, only one study has investigated the evaluation of PFVP in coronary artery disease (CAD) patients.^25^ This experimentation conducted by our research group compared PFVP after two 8-s sprints on a cycle ergometer between CAD patients and age-matched healthy adults.^25^ We found that CAD patients had a lower force production at both low and high velocities. Moreover, we also showed that CAD patients tolerated this type of effort. Therefore, assessing the PFVP on a cycle ergometer at the beginning of CR, in order to tailor physical training to each patient, potentially may lead to better results than the usual ‘one-size-fits-all’ CR programme.

The aim of this study was to compare the effects of two exercise training programmes in CAD patients: a traditional CR vs. a new CR based on patients’ individual PFVP on cardiorespiratory, biological, and muscular systems. We hypothesized that the novel CR would be more effective than conventional CR for VO_2_ at VT_1_ and VO_2 peak_, primary and secondary endpoints, respectively. We assumed that both groups would obtain similar benefits on the lipid profile and muscle variables.

## Methods

### Study population

This study was conducted in a single rehabilitation centre (Saint-Etienne University Hospital, Saint-Etienne, France). Eligible criteria were (i) to have acute coronary syndrome treated within the last 6 months, treated with coronary revascularization by percutaneous coronary intervention (angioplasty with stent implantation) or surgery (coronary artery bypass grafting); (ii) to be over 18 years old; (iii) to be clinically stable; (iv) to have a maximal aerobic power (MAP) on cycle ergometer > 60 W for women and 80 W for men during the cardiopulmonary exercise testing (CPET).^3,26^ Participants were excluded if they reported significant cardiac, respiratory, or joint comorbidities preventing high-intensity physical training.

Written informed consent was obtained from all participants. The study was in accordance with the Declaration of Helsinki and was approved by an Institutional Review Board (2018-A01613-52). The trial was registered at ClinicalTrials.gov (NCT04102410).

### Experimental design

**Figure 1:**
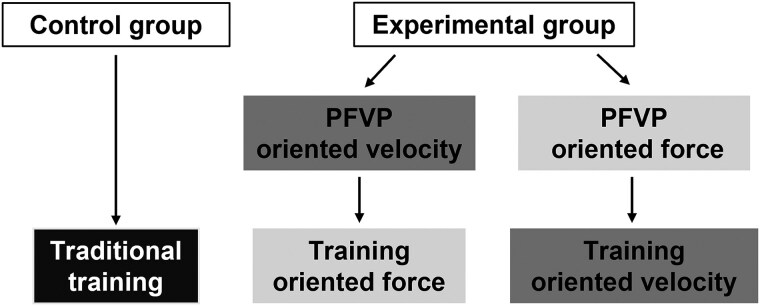
Physical training according to participants of each group. PFVP, power–force–velocity profile.

This was a prospective, controlled, open-label, and randomized trial. Eligible patients were randomized in a 1:1 ratio to two groups: control or experimental. The control group followed a conventional CR, while the training programme of the experimental group was oriented in force or in velocity according to the initial PFVP profile of the participants (*Figure 1*).

Cardiac rehabilitation was offered eight days after percutaneous intervention and 21 days after coronary artery bypass grafting. Patients benefited from this multidisciplinary medical and paramedical care for 3 weeks. Each CR group included six patients. Participants completed four physical activity sessions of 2 hours per week. The last day of the week was devoted to lifestyle therapeutic education.

### Physiological measurements

#### Cardiorespiratory

The primary and secondary endpoints were VO_2_ at VT_1_ and VO_2_  _peak_, respectively. These variables were obtained by a maximum CPET. The same electronically braked cycle ergometer and procedures were used as in a previous original research.^27^ VO_2_  _peak_ was defined as the mean value of the last 30 s of exercise. MAP and respiratory exchange ratio (RER) were also measured at the end of exertion. Cardiac output (Q_c max_) and stroke volume (VES_max_) at VO_2_  _peak_ were measured using a thoracic bioelectrical impedance device (PhysioFlow® PF07 Enduro™, Paris, France). The PhysioFlow device and methodology have been thoroughly described elsewhere.^28^ Participants were monitored continuously with a 12-lead electrocardiogram. Hence, the heart rate (HR) was recorded. Systolic blood pressure (BP) was measured manually by an experienced nurse using a random-zero sphygmomanometer when the participant was sitting on the cycle ergometer at rest and at the peak of exercise.

#### Biological

Blood samples were performed to check lipid levels [total cholesterol, HDL and LDL cholesterol (LDL-C), and triglycerides].

#### Functional and muscular

Short-form 12 questionnaire was used to estimate participants’ quality of life.

Handgrip force was evaluated using a Saehan hydraulic hand dynamometer (Model SH5001, Saehan Corporation, Changwon, South Korea). The patient was in a standing position, with the dominant arm stretched out in front and performed three maximum pressures of 3–4 s separated by 1 min of rest. The most important force of the three trials was retained.

Quadriceps isometric muscle strength was assessed using an ergometer chair equipped with a force sensor (LegControl, MTraining®, Ecole-Valentin, France). Participants were positioned at 90° of hip and knee flexions with their arms crossed over their chest. They had to push with their dominant leg as hard as possible against the force lever placed at their ankle. They performed three leg extensions of 3–4 s, separated by 1 min of rest. As with the handgrip test, the best of three trials was saved.

These physiological data were collected during the week before and after the CR programme.

### Determination of the power–force–velocity profile

**Figure 2:**
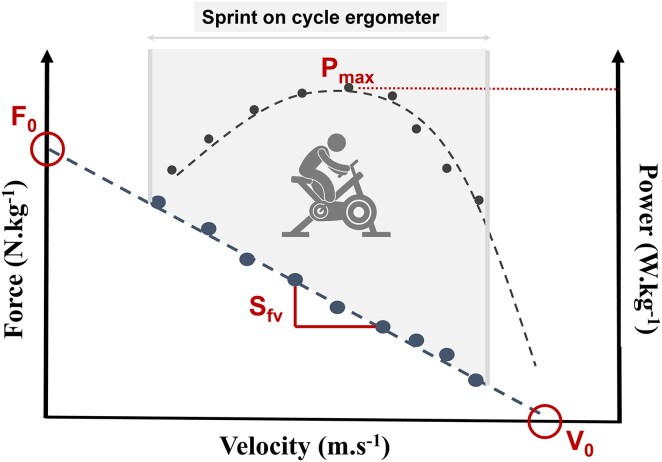
Main parameters of the power–force–velocity profile. F_0_, theoretical maximum force; P_max_, maximal power output; S_fv_, slope of the linear force–velocity relationship; V_0_, theoretical maximum velocity.

Patients performed two sprints of 8 s on a cycle ergometer (Monark, Vansbro, Sweden) to obtain their PFVP on the first and the last days of CR, after a 5-min warm-up with increasing intentional velocity. Friction loads were set at 0.4 and 0.3 N·kg^−1^ for men and women, respectively, based on preliminary testing and a pilot study. All features of the ergometer and the same procedures to assess PFVP were used and described in the pilot study.^25^ Key parameters of the PFVP were calculated: the maximal power output (P_max_), the theoretical maximum force (F_0_), the theoretical maximum velocity (V_0_), and the slope of linear FV relationship (S_fv_) (*Figure 2*). We focused on the S_fv_ to determine the patient’s PFVP and his deficient quality (see Supplementary material online, *Material S1* for more details).

### Physical exercise programme

**Figure 3:**
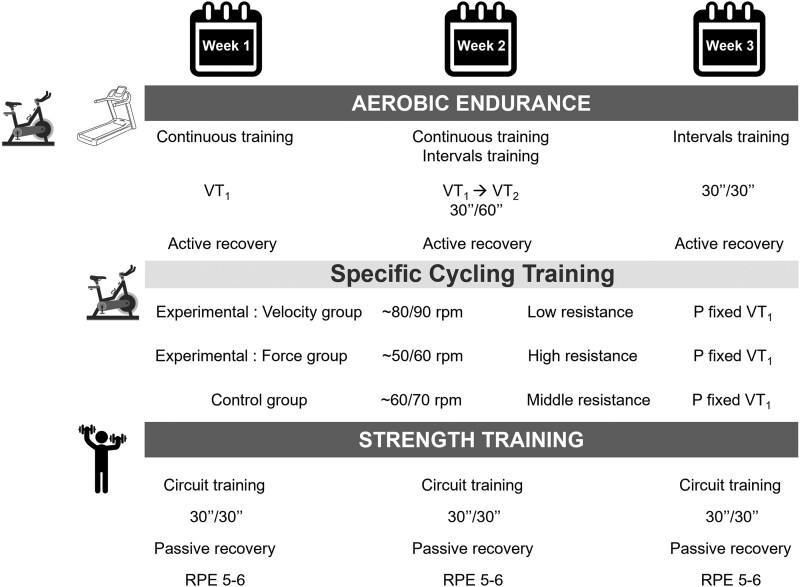
Physical exercise intervention. P, power; RPE, rate of perceived exertion; rpm, rotations per minute; VT, ventilatory threshold.

The physical training programme is illustrated in *Figure 3*. Each exercise session consisted of a warm-up, a cardiovascular training period, and a cool-down phase. Patients wore a HR monitor belt throughout the physical session. Aerobic endurance represented the majority of the CR programme since each training session included at least 40 min on the cycle ergometer and 10 min on the treadmill. The intensity of this prolonged submaximal exercise was adapted according to the CPET results of each patient, more precisely HR and power at VT_1_ and the peak of exercise.^3^ However, in this original study, we relied on these results as well as on the patient's PFVP.

The experimental group was trained according to less developed quality as assessed by PFVP. They followed a specific training oriented on force or on velocity on an exercise bike (Laroq CMVC16, flywheel mass: 7 kg, MTraining®, Ecole-Valentin, France). The power of the cycle ergometer was fixed and adapted to the patient’s capacities at VT_1_. We asked the patients to maintain a specific pedalling cadence; therefore, the resistance of the training bike was automatically adapted. More specifically, continuous training focussed on force was characterized by low pedalling cadence (between 50 and 60 rpm) and high resistance. Conversely, the training oriented in velocity was defined by a high pedalling cadence (between 80 and 90 rpm) and lower resistance. A conventional training (i.e. an intermediate between 60 and 70 rpm) was proposed to the control group. In addition, an aerobic interval training in 5- to 10-min sequences was gradually offered as an alternative to continuous endurance exercise.

Participants benefitted from the same training content regarding aerobic endurance on a treadmill and in dynamic resistance independently of their group. The dynamic resistance training was offered twice a week and consisted of an overall muscle strength training, such as sheathing exercises or focussed more on the lower or upper limbs.

### Therapeutic education

Participants also received four therapeutic education sessions by various health professionals covering mainly disease knowledge, nutrition, risk factors, and pharmacological treatment.

### Statistical analysis

Statistical analyses were performed using JASP (version 0.16.3). To assess the difference between the two groups at baseline, we used a *t*-test for quantitative variables and a *χ*² test for qualitative parameters. The effect of the training programme on physiological parameters according to the CR method was evaluated using two-way repeated measures analysis of variance (ANOVA), i.e. CR groups (experimental vs. control) × time (pre–post). The ANOVA was adjusted for relevant significant variables between the two groups at baseline. Where a significant interaction difference occurred, Tukey’s *post hoc* analyses were performed. Effect size was calculated only for significant findings. All data were reported as mean ± standard deviation (SD). For all statistical comparisons, the level of significance was set at *P* < 0.05.

## Results

**Figure 4:**
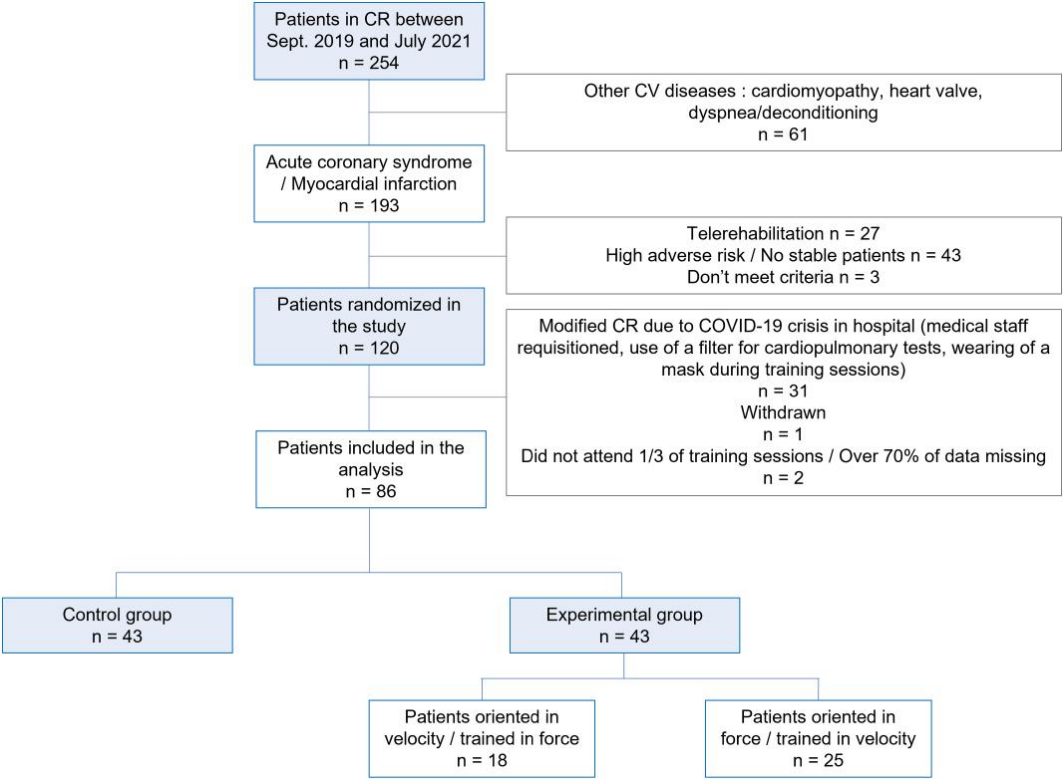
Decision flowchart. CR, cardiac rehabilitation; CV, cardiovascular; Sept, September.

A total of 86 patients (mean age: 60.8 ± 9.6 years; 15% were women) were equally allocated in both groups (*Figure 4*). In the experimental group, 18 patients followed specific training in force and 25 in velocity (for more details, see *Figure S1*). No adverse events occurred during the CR period, and only one participant did not complete the exercise intervention.

Cardiovascular risk factors, cardiac intervention, medical treatment, and baseline characteristics of both groups are described in *Table 1*. Forty-four patients (98%) had at least one cardiovascular risk factor, and 54 (63%) were overweight [body mass index (BMI) > 25 kg·m^−2^]. Body mass, BMI, coronary revascularization, and the percentage of diabetic patients were statistically different between both groups at baseline (*Table 1*).

**Table 1:** Descriptive of patients’ characteristics

Variable	Control group (*n* = 43)	Experimental group (*n* = 43)	*P*-value
Age (years)	60.2 ± 9.8	61.3 ± 9.5	0.586
Females	9 (21)	4 (9)	0.132
**Body mass (kg)**	**73.9** **±** **15.3**	**83.4** **±** **12.8**	**0**.002**
**BMI (kg·m^−2^)**	**25.8** **±** **4.7**	**28.3** **±** **4.2**	**0**.014*
Sport	12 (28)	8 (19)	0.307
Risk factors			
Family history	11 (26)	16 (37)	0.245
Personal CV history	7 (16)	4 (9)	0.500
**Diabetes mellitus**	**3 (7)**	**11 (26)**	**0**.019*
Hyper blood pressure	14 (33)	19 (44)	0.268
Dyslipidaemia	11 (26)	12 (28)	0.808
Psychological disorders	15 (35)	8 (19)	0.088
Sleep apnoea	10 (23)	13 (30)	0.465
Tobacco consumption			0.335
Never or former	28 (65)	21 (49)	
Stop after cardiac event	10 (23)	17 (40)	
Current	5 (12)	5 (12)	
Medical treatment			
Aspirin	43 (100)	42 (98)	0.314
DAPT	41 (95)	42 (98)	0.557
Beta-blockers	39 (91)	39 (91)	1.000
ACEI/ARB	39 (91)	41 (95)	0.397
Statins	41 (95)	40 (93)	0.645
Cardiac function and intervention			
LVEF (%)	53.6 ± 9.6	57.3 ± 8.0	0.067
Coronary artery intervention			0.027*
Angioplasty (stenting)	36 (84)	42 (98)	
CABG	7 (16)	1 (2)	

Values are expressed as mean ± SD or *n* (%). Significant difference between groups: *P < 0.05; **P < 0.01. Bold values represent only data with a statistically significant difference.

ACEI/ARB, angiotensin converting enzyme inhibitor/angiotensin receptor blocker; BMI, body mass index; CABG, coronary artery bypass grafting; CV, cardiovascular; DAPT, double antiplatelet; LVEF, left ventricular ejection fraction.

Significant descriptive variables were included as covariates in the statistical model. The results are presented in *Table 2*. All other secondary parameters measured are described and reported in Supplementary material online, *Material S2* and *Table S1*, respectively.

**Table 2:** Effect of 3-week specific and traditional exercise training in cardiac rehabilitation programme based on physiological and mechanical parameters

Variable	Control group		Experimental group	
	Pre	Post	Pre	Post
**Cardiorespiratory and vascular**				
**VO_2_ at VT_1_ (mL·min^−1^**·**kg^−1^)**	12.8 ± 3.3	13.5 ± 4.1***	12.7 ± 3.2	16.4 ± 3.4***^a^
**VO_2_ _peak_ (mL·min^−1^**·**kg^−1^)**	19.4 ± 4.6	21.3 ± 5.6***	20.8 ± 4.4	24.8 ± 4.4***^,a,b^
**MAP (W)**	110.1 ± 32.0	129.1 ± 35.5	134.9 ± 36.7	165.0 ± 37.4^a,b^
**RER**	1.12 ± 0.10	1.12 ± 0.12	1.10 ± 0.09	1.14 ± 0.10^a^
**Q_c max_ (L**·**min^−1^)**	13.8 ± 4.3	14.2 ± 3.8	14.0 ± 3.1	16.1 ± 3.5
**VES_max_ (mL)**	114.8 ± 28.8	115.9 ± 27.9	122.5 ± 28.3	128.0 ± 29.8
**HR (bpm)**				
**Rest**	69.6 ± 10.0	70.6 ± 15.4	69.5 ± 14.0	68.3 ± 13.6
**Max**	118.3 ± 18.0	127.8 ± 24.5	124.7 ± 22.0	132.4 ± 20.1
**Syst BP (mmHg)**				
**Rest**	126.6 ± 20.0	122.1 ± 17.5	125.5 ± 17.2	121.4 ± 17.7
**Max**	166.7 ± 24.3	171.7 ± 29.4	175.0 ± 26.9	185.2 ± 31.8
**Biological**				
**TC (g**·**L^−1^)**	1.27 ± 0.29	1.26 ± 0.25	1.32 ± 0.35	1.23 ± 0.33
**LDL-C (g**·**L^−1^)**	0.63 ± 0.19	0.61 ± 0.18	0.68 ± 0.29	0.56 ± 0.21^a^
**HDL-C (g**·**L^−1^)**	0.41 ± 0.09	0.44 ± 0.11*	0.42 ± 0.10	0.45 ± 0.12*
**TGL (g**·**L^−1^)**	1.10 ± 0.53	1.01 ± 0.39	1.28 ± 0.92	1.10 ± 0.83
**Functional and muscular**				
**HRQoL**	91.3 ± 14.2	97.4 ± 12.3	90.2 ± 12.1	98.9 ± 8.8
**Handgrip force (N**·**kg^−1^)**	4.6 ± 1.3	5.0 ± 1.5***	4.9 ± 1.1	5.2 ± 0.9***
**Quadriceps force (N**·**kg^−1^)**	4.4 ± 1.5	4.8 ± 1.6***	4.7 ± 1.2	5.2 ± 1.2***

Significant differences with PRE: *P < 0.05; **P < 0.01; ***P < 0.001.

HDL-C, HDL cholesterol; HR, heart rate; HRQoL, health-related quality of life; LDL-C, LDL cholesterol; MAP, maximum aerobic power; Q_c_  _max_, maximum cardiac output; RER, respiratory exchange ratio; syst BP, systolic blood pressure; TC, total cholesterol; TGL, triglycerides; VES_max_, maximum systolic ejection volume; VO_2_ at VT_1_, oxygen uptake at the first ventilatory threshold; VO_2_ peak, peak oxygen uptake.

^a^Significant interaction effect (time × group).

^b^Significant differences with control group.

**Figure 5:**
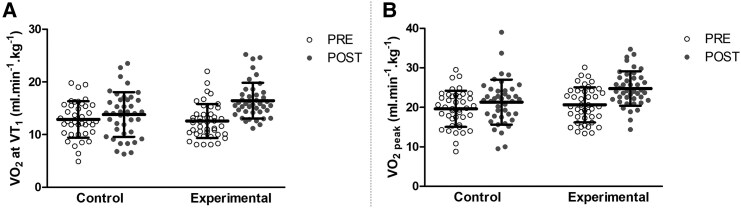
Effect of 3-week specific and traditional exercise training on the oxygen uptake at the first ventilatory threshold (panel A) and on the peak oxygen uptake (panel B). VO_2_ at VT_1_, oxygen uptake at the first ventilatory threshold; VO_2_ peak, peak oxygen uptake.

*Figure 5* illustrates the increase of VO_2_ at VT_1_ and VO_2_  _peak_ at the end of the training CR programme in both groups (time effect: VO_2_ at VT_1_, *P* < 0.001, *η_p_*^2^ = 0.31 and VO_2_  _peak_, *P* < 0.001, *η_p_*^2^ = 0.44). Nevertheless, the gain was significantly higher in the experimental group (VO_2_ at VT_1_: +3.8 ± 3.3 mL·min^−1^·kg^−1^; VO_2_  _peak_: +4.0 ± 3.3 mL·min^−1^·kg^−1^) than in the control group (VO_2_ at VT_1_: +0.74 ± 3.6 mL·min^−1^·kg^−1^; VO_2_  _peak_: +1.9 ± 3.4 mL·min^−1^·kg^−1^) for both parameters (interaction effect: VO_2_ at VT_1_, *P* < 0.001, *η_p_*^2^ = 0.17 and VO_2_  _peak,_  *P* < 0.01, *η_p_*^2^ = 0.09). An interaction effect was also found in RER (*P* = 0.049, *η_p_*^2^ = 0.05) and MAP (*P* = 0.004, *η_p_*^2^ = 0.10). Like VO_2_ measurements, a better benefit was obtained in the experimental group (RER: +4.1 ± 6.5%; PMA: +24.6 ± 15.2%) than in the control group (RER: +1.0 ± 10.1%; PMA: +18.8 ± 16.9%). Indeed, Tukey’s *post hoc* revealed greater gains in the experimental group than in the control group. Furthermore, a significant difference between groups for VO_2_  _peak_ and MAP was found. No significant effects were observed at the end of the experimentation for Q_c max_, VES_max_, HR, and systolic BP.

For biological measurements, HDL cholesterol (HDL-C) (*P* = 0.017, *η_p_*^2^ = 0.09) improved after a 3-week intervention in both study populations (experimental: +8.6 ± 20.0%; control: +7.9 ± 11.2%). Surprisingly, we found a greater decrease of LDL-C (interaction effect: *P* = 0.01, *η_p_*^2^ = 0.11) in the experimental group (−0.02 ± 0.09 g·L^−1^) than in the control group (−0.12 ± 0.27 g·L^−1^). No difference was noted for CT and TG in the two groups.

An increase of isometric muscle force of handgrip (time effect: *P* < 0.001, *η_p_*^2^ = 0.21) and quadriceps (time effect: *P* < 0.001, *η_p_*^2^ = 0.59) was observed after CR independently of the group. We noticed no significant change of quality of life perception.

## Discussion

The European Society of Cardiology recommends that CR should be tailored to the patient.^3,5^ Regarding exercise training, most studies have focused on training modalities (duration, frequency, and intensity) in particular types of training (continuous or interval trainings).^15^ The mode of muscle contraction has also been investigated (concentric vs. eccentric).^29^ More recently, a study has compared centre-based and home-based CR.^27^ To our knowledge, the current study is the first to propose a personalized exercise programme according to the cardiorespiratory and neuromuscular mechanical outputs of the patients.

All muscle variables, as well as HDL-C and VO_2_ measurements, were enhanced at the end of the CR programme. The main and the secondary evaluation criteria, VO_2_ at VT_1_ and VO_2_  _peak_, increased to a greater extent in the experimental group compared with the control group.

Our main hypothesis was that this individualized training would result in a greater increase of VO_2_ at VT_1_ and VO_2 peak_ compared with the conventional CR. We demonstrated that physical training optimized by PFVP was more effective on both these cardiorespiratory parameters. VO_2_  _peak_ that is one of the strongest markers of physical capacities improved after the 3-week CR programme in both study populations. Indeed, this key cardiorespiratory indicator increased by 11% and 22% in the control and experimental groups, respectively. The positive change in VO_2_  _peak_ values was similar to those obtained in previous studies.^30,31^

However, a recent investigation compared generic and individualized power training based on PFVP in older adults (mean age: 68 ± 5 years).^32^ Conversely, the authors found that a personalized programme did not improve physical function further than a generic power training. This finding could be partly explained by a difference in PFVP assessment and determination (pneumatic resistance on leg press vs. friction load on cycle ergometer in our study), training modality (strength training with loads vs. cycling aerobic programme in our research), and high-force or velocity content actually reached during training, training frequency and duration (10-week with two sessions per week vs. 3-week period with four sessions per week in our study), outcome measures, adaptations, and especially population characteristics (older adults and CAD patients).

More individualized training focused on force or on velocity may have induced greater cardiovascular and neuromuscular adaptations. The increase in VO_2_ peak depends on multiple factors like Q_c max_ and maximum difference in arteriovenous O_2_ (AVO_2_) content (Fick’s equation).^30^ We did not observe any significant difference for HR_max_ and VES_max_ parameters. Hence, from a mathematical point of view, it seems likely that the significant difference between baseline and after CR programme in VO_2_  _peak_ was influenced by AVO_2_. Moreover, we found a higher VO_2_  _peak_ in the experimental group than in the control group. We did not notice any interaction (time × group) effect in Q_c max_, VES_max_, syst BP _max_, HR_max_, so we could speculate that the improvement of VO_2 peak_ in the interventional group was also related to vascular and peripheral neuromuscular adaptations. Therefore, the highest VO_2_  _peak_ could be related to peripheral vascular adaptations through better O_2_ transport and utilization in skeletal muscle, a higher peripheral vasodilatation and capillary density, and mitochondrial adaptations.^33^ Given the short training period, different neuromuscular mechanisms could also be responsible such as an enhanced muscular coordination and better nervous activation patterns (synchronization and recruitment of motor units, activation fast-twitch muscle fibres).^34–36^ Finally, it is noteworthy that we had a greater proportion of responders in the experimental group (86%) than in the control group (70%) on the primary evaluation criterion (i.e. VO_2_ at VT_1_). This strategy may thus be helpful to enhance the rate of responders to exercise training, compared with conventional CR. Furthermore, this training based on PFVP considered the mechanical characteristics of each patient and was adapted to their needs, unlike conventional common training that leads to positive changes for some participants and negative for others. To assert our last statement, we noted more responders.

Concerning blood tests, HDL-C improved at the end of a 3-week physical intervention in the two groups. We speculated that the physical activity practiced in CR and the optimization of nutrition through therapeutic education interventions have contributed to this benefit.^37,38^ An interesting and surprising result was that patients who followed a more individualized training achieved a greater reduction in LDL-C compared with patients trained with a conventional approach. Indeed, the LDL-C decreased by 15.5% and by 0.8% in the experimental and control groups, respectively. Moreover, patients in the group trained according to their initial PFVP almost reached the recommended standard for secondary prevention (<55 mg·dL^−1^).^5,37^ LDL-C levels are modified by diet, regular statin therapy, body weight changes, and physical activity.^37^ We hypothesized that the specific training had a better positive impact than other factors. Since there were no significant differences between groups in medical treatment, all patients participated in the same nutritional workshop. We only found a significant difference in body mass between groups in favour of the control group.

Both study populations enhanced muscle force of upper and lower limbs. This outcome can be explained by a common, non-personalized resistance training programme. More specifically, the content of resistance training sessions was the same for all participants.

Although this is the first investigation to optimize the training programme in CR to the individual mechanical characteristics of the PFVP, some limitations should be discussed. The fixed training duration of 3 weeks may not have been ideal for all participants. This programme may be more suited to young CAD patients because it is shorter than the usual CR programmes, so patients could return to work earlier. These favourable results could be confirmed with a larger number of volunteers in subgroups. It is recommended to use two distant friction loads to determine the PFVP on cycling.^39,40^ However, it would not be appropriate to apply a load corresponding to more than 50% of the CAD patient's body mass. Other measurements would also be relevant to better understand the training responses and neurophysiological adaptations of these different exercise programmes. For instance, it would be relevant to characterize the influence of the rating of perceived exertion on exercise tolerance.^41,42^

In conclusion, the training rehabilitation programme resulted in a significant improvement in mechanical and cardiorespiratory capacities and lipid status in both groups. With the innovative training based on the patient's PFVP, the experimental group achieved better improvements than the control group on VO_2_ at VT_1_, VO_2_  _peak_, and LDL-C. Hence, PFVP could be used in CR to adapt the content of a physical training session for each patient. In addition to the feasibility and better health benefits, tailoring CR to each patient may be improve cost-effectiveness and patient adherence in long term.

## Supplementary Material

oeaf036_Supplementary_Data

## Data Availability

The original contributions presented in the study are included in the article/Supplementary material; further inquiries can be directed to the corresponding author.
