# Supplementary material for: An innovative cardiac rehabilitation based on the power–force–velocity profile to further improve cardiorespiratory capacities in coronary artery disease patients: CITIUS study
Source: Eur Heart J Open. 2025 Apr 22;5(3):oeaf036. doi: 10.1093/ehjopen/oeaf036 (PMC12152306; doi:10.1093/ehjopen/oeaf036)
Supplement: oeaf036_Supplementary_Data [file oeaf036_supplementary_data.zip › Supplementary_Material_Online_S1.docx]

Definition of the power-force-velocity profile orientation

On the first and the last days of CR, after a 5-min warm-up with increasing intentional velocity, patients performed 2 sprints of 8-s on a cycle ergometer (Monark, Vansbro, Sweden) to obtain their PFVP. Friction loads were set at 0.4 N/kg and 0.3 N/kg for men and women, respectively. All features of the ergometer and the same procedures to assess PFVP were used and described in the pilot study.^25^ Key parameters of the PFVP were measured: the maximal power output (P_max_), the theoretical maximum force (F_0_), the theoretical maximum velocity (V_0_), and the slope of linear FV relationship (S_fv_) (Figure 2).

To determine whether the patient had a profile oriented in force or in velocity, we analyzed the different parameters of the PFVP. We focused on S_fv_, as this variable illustrates the athlete's individual balance between force and velocity abilities. Through previous experiments, we have collected a large number of PFVP in healthy adults (N=321) with different characteristics (age, sex, physical activity level). The healthy inactive adults (N=75, 40% women, age=59 ± 13 y) were separated in two subgroups according to sex. Then, linear regressions between age and S_fv_ were made for both men (y=0.003x – 5.0217) and women (y=0.0226x – 5.3749).

This linear regression has become our standard. The patients’ profile was defined in force or in velocity depending on if their slope value was, respectively, above or below the regression line. For example, if the patient's data lies above the mean regression, we deduce that his deficient quality is velocity, since his PFVP is oriented in force.
