# Supplementary material for: An innovative cardiac rehabilitation based on the power–force–velocity profile to further improve cardiorespiratory capacities in coronary artery disease patients: CITIUS study
Source: Eur Heart J Open. 2025 Apr 22;5(3):oeaf036. doi: 10.1093/ehjopen/oeaf036 (PMC12152306; doi:10.1093/ehjopen/oeaf036)
Supplement: oeaf036_Supplementary_Data [file oeaf036_supplementary_data.zip › Supplementary_Material_Online_S2.docx]

*Physiological measurements*

An initial transthoracic echocardiography was carried out to define the left ventricular ejection fraction. Patients repeated this test only if an abnormality was detected the first time.

**Cardiorespiratory.** The primary and secondary endpoints were VO_2_ at VT_1_ and VO_2_ _peak_, respectively. These variables were obtained by a maximum CPET. The same electronically braked cycle ergometer and procedures were used as in a previous original research.^27^ VO_2_ _peak_ was defined as the mean value of the last 30 s of exercise. MAP and respiratory exchange ratio (RER) were also measured at the end of exertion. Cardiac output (Q_c max_) and stroke volume (VES_max_) at VO_2_ _peak_ were measured using a thoracic bioelectrical impedance device (PhysioFlow^®^ PF07 Enduro^TM^, Paris, France). The PhysioFlow device and methodology have been thoroughly described elsewhere.^28^ Participants were monitored continuously with a 12-lead electrocardiogram (ECG). Hence, the heart rate (HR) was recorded. Systolic blood pressure (BP) was measured manually by an experienced nurse using a random-zero sphygmomanometer when the participant was sitting on the cycle ergometer at rest, every 2 min during exercise, and at 1-min recovery from exercise.

**Biological.** Blood samples were performed to check glycemic (fasting blood glucose) and lipid levels (total cholesterol, HDL and LDL cholesterol, and triglycerides).

**Muscular.** Handgrip force was evaluated using a Saehan hydraulic hand dynamometer (Model SH5001, Saehan Corporation, Changwon, South Korea). The patient was in a standing position, with the dominant arm stretched out in front and performed three maximum pressures of 3-4 s separated by 1 min of rest. The most important force of the three trials was retained.

Quadriceps isometric muscle strength was assessed using an ergometer chair equipped with a force sensor (LegControl, MTraining^®^, Ecole-Valentin, France). Participants were positioned at 90° of hip and knee flexions with their arms crossed over their chest. They had to push with their dominant leg as hard as possible against the force lever placed at their ankle. They performed three leg extensions of 3-4 s, separated by 1 min of rest. As with the handgrip test, the best of three trials was saved.

**Autonomic.** Heart rate variability (HRV) indices were measured from 24-h ECG Holter recordings (Vista, Novacor, Rueil-Malmaison, France) using HRV analysis free software. Time domain indices were calculated as SDNN (standard deviation of normal R-R intervals), RMSSD (square root of the mean squared differences of successive R-R intervals), and pNN50 (percentage of differences between adjacent normal R-R intervals >50 ms). Fourier transform characterises a signal by its frequency spectrum. This analysis allows us to highlight different frequency ranges: low frequency (LF) and high frequency (HF). The LF/HF ratio allows to distinguish the balance between the sympathetic and the parasympathetic nervous systems.

Spontaneous relative variations in BP and R-R interval duration were recorded by measuring simultaneously a plethysmography BP and ECG (Finapres Medical Systems BV, Netherlands) over a 15-min period. The patient was in supine position at rest. The increase or decrease in BP and the corresponding lengthening or shortening response of the following R-R interval was analysed. Spontaneous sequences of at least three consecutive beats characterised by a progressive increase in BP followed by a change in R-R interval length were automatically detected (R-R per mmHg). Hence, the linear regressions were plotted at each event. The average slope of these regressions was considered as the index of sensitivity of arterial baroreflex (BRS).

These physiological data were collected during the week before and after the CR programme.
