# Supplementary material for: An innovative cardiac rehabilitation based on the power–force–velocity profile to further improve cardiorespiratory capacities in coronary artery disease patients: CITIUS study
Source: Eur Heart J Open. 2025 Apr 22;5(3):oeaf036. doi: 10.1093/ehjopen/oeaf036 (PMC12152306; doi:10.1093/ehjopen/oeaf036)
Supplement: oeaf036_Supplementary_Data [file oeaf036_supplementary_data.zip › Table_S1.docx]

| Variable | Control Group | | Experimental Group | |  |
| --- | --- | --- | --- | --- | --- |
|  | PRE | POST | PRE | POST |  |
| *Anthropometry* | | | | | |
| Body mass (kg) | **73.9 ± 15.3** | **74.1 ± 15.6** | **83.4 ± 12.8** | **83.2 ± 13.2‡** |  |
| BMI (kg.m^-2^) | **25.8 ± 4.7** | **25.8 ± 4.8** | **28.3 ± 4.2** | **28.1 ± 4.2‡** |  |
| Waist circumference (cm) | **93.8 ± 12.4** | **93.2 ± 12.3** | **100.6 ± 12.3** | **100.4 ± 13.2‡** |  |
| Hip circumference (cm) | **99.0 ± 10.8** | **98.0 ± 10.5*** | **103.7 ± 8.5** | **101.7 ± 11.9*** |  |
| Thigh circumference (cm) | **47.7 ± 5.6** | **47.0 ± 5.8** | **49.7 ± 4.2** | **49.9 ± 4.1‡** |  |
| *Cardiorespiratory & Vascular* | | | | | |
| VO_2_ at VT_1_  (ml.min^-1^.kg^-1^) | **12.8 ± 3.3** | **13.5 ± 4.1***** | **12.7 ± 3.2** | **16.4 ± 3.4***†** |  |
| VO_2_ _peak_  (ml.min^-1^.kg^-1^) | **19.4 ± 4.6** | **21.3 ± 5.6***** | **20.8 ± 4.4** | **24.8 ± 4.4***†‡** |  |
| VO_2 peak_  (theoretical %) | **73.1 ± 11.9** | **81.0 ± 16.1***** | **83.3 ± 17.6** | **99.3 ± 16.0***†‡** |  |
| Power at VT1 (W) | **58.3 ± 20.9** | **74.1 ± 26.7***** | **71.3 ± 25.1** | **96.6 ± 30.2***‡** |  |
| MAP (W) | **110.1 ± 32.0***** | **129.1 ± 35.5** | **134.9 ± 36.7** | **165.0 ± 37.4***†‡** |  |
| Pulse O2 | **12.5 ± 4.0** | **13.0 ± 4.5***** | **14.8 ± 3.4** | **16.4 ± 3.4***†‡** |  |
| RER | **1.12 ± 0.10** | **1.12 ± 0.12*** | **1.10 ± 0.09** | **1.14 ± 0.10*†** |  |
| PWV (m.s^-1^) | 10.0 ± 5.3 | 9.1 ± 4.4 | 9.8 ± 4.4 | 10.1 ± 2.8 |  |
| Q_c max_ (L.min^-1^) | 13.8 ± 4.3 | 14.2 ± 3.8 | 14.0 ± 3.1 | 16.1 ± 3.5 |  |
| VES_max_ (mL) | 114.8 ± 28.8 | 115.9 ± 27.9 | 122.5 ± 28.3 | 128.0 ± 29.8 |  |
| HR (bpm): | | | | | |
| rest | 69.6 ± 10.0 | 70.6 ± 15.4 | 69.5 ± 14.0 | 68.3 ± 13.6 |  |
| max | 118.3 ± 18.0 | 127.8 ± 24.5 | 124.7 ± 22.0 | 132.4 ± 20.1 |  |
| 1-min recovery | 104.7 ± 17.3 | 111.5 ± 23.8 | 107.6 ± 18.6 | 113.0 ± 18.7 |  |
| 3-min recovery | 91.8 ± 13.2 | 90.8 ± 16.6 | 91.4 ± 17.5 | 91.9 ± 15.4 |  |
| BP syst (mmHg): | | | | | |
| rest | 126.6 ± 20.0 | 122.1 ± 17.5 | 125.5 ± 17.2 | 121.4 ± 17.7 |  |
| max | 166.7 ± 24.3 | 171.7 ± 29.4 | 175.0 ± 26.9 | 185.2 ± 31.8 |  |
| 1-min recovery | 163.1 ± 33.3 | 169.4 ± 34.0 | 174.9 ± 28.8 | 187.2 ± 36.3 |  |
| 3-min recovery | 153.1 ± 29.8 | 155.6 ± 26.7 | 163.4 ± 26.4 | 171.3 ± 27.7 |  |
| BP diast (mmHg): | | | | | |
| rest | 76.9 ± 15.7 | 72.6 ± 11.3 | 75.3 ± 13.4 | 74.0 ± 12.7 |  |
| max | 79.3 ± 15.4 | 78.3 ± 15.4 | 80.8 ± 13.9 | 80.1 ± 12.6 |  |
| 1-min recovery | 77.4 ± 15.4 | 79.0 ± 18.5 | 77.0 ± 15.2 | 77.7 ± 14.2 |  |
| 3-min recovery | 74.5 ± 15.2 | 75.1 ± 13.2 | 74.3 ± 14.5 | 76.1 ± 13.4 |  |
| *Functional & Muscular* | | | | | |
| 6WT (m) | **444.9 ± 79.3** | **491.1 ± 81.9***** | **477.8 ± 80.3** | **533.5 ± 83.8*******‡** |  |
| Sit to stand (s) | **25.8 ± 6.0** | **21.3 ± 4.5***** | **23.9 ± 6.2** | **19.6 ± 6.1***** |  |
| HRQoL | 91.3 ± 14.2 | 97.4 ± 12.3 | 90.2 ± 12.1 | 98.9 ± 8.8 |  |
| Handgrip force (N.kg^-1^) | **4.6 ± 1.3** | **5.0 ± 1.5***** | **4.9 ± 1.1** | **5.2 ± 0.9***** |  |
| Quadriceps force (N.kg^-1^) | **4.4 ± 1.5** | **4.8 ± 1.6***** | **4.7 ± 1.2** | **5.2 ± 1.2***** |  |
| *Biological* | | | | | |
| Fasting blood glucose (g.L^-1^) | 1.09 ± 0.40 | 1.13 ± 0.48 | 1.14 ± 0.30 | 1.09 ± 0.21 |  |
| TC (g.L^-1^) | 1.27 ± 0.29 | 1.26 ± 0.25 | 1.32 ± 0.35 | 1.23 ± 0.33 |  |
| LDL-C (g.L^-1^) | **0.63 ± 0.19** | **0.61 ± 0.18***** | **0.68 ± 0.29** | **0.56 ± 0.21***†** |  |
| HDL-C (g.L^-1^) | **0.41 ± 0.09** | **0.44 ± 0.11***** | **0.42 ± 0.10** | **0.45 ± 0.12***** |  |
| TGL (g.L^-1^) | **1.10 ± 0.53** | **1.01 ± 0.39**** | **1.28 ± 0.92** | **1.10 ± 0.83**** |  |
| *Autonomic* | | | | | |
| SDNN (ms) | 115.7 ± 35.6 | 126.7 ± 33.5 | 124.7 ± 44.3 | 122.2 ± 45.3 |  |
| RMSSD (ms) | 40.2 ± 30.4 | 42.5 ± 41.5 | 34.4 ± 17.1 | 38.6 ± 21.1 |  |
| pNN50 (%) | 8.7 ± 9.3 | 7.9 ± 10.4 | 7.9 ± 9.6 | 9.3 ± 10.5 |  |
| BRS (ms.mmHg^-1^) | 8.8 ± 6.7 | 10.3 ± 13.1 | 6.7 ± 4.1 | 9.3 ± 8.5 |  |
| LF (m²/Hz) | 319.2 ± 481.3 | 694.1 ± 2353.7 | 269.8 ± 420.5 | 361.5 ± 650.0 |  |
| HF (m²/Hz) | 199.5 ± 289.4 | 363.4 ± 1204.6 | 165.2 ± 284.6 | 308.0 ± 543.2 |  |
| LF/HF ratio | 2.09 ± 1.92 | 2.33 ± 1.45 | 3.00 ± 2.86 | 2.34 ± 1.65 |  |
| *Force-velocity profile* | | | | | |
| F_0_ (N.kg^-1^) | **1.20 ± 0.26** | **1.27 ± 0.30***** | **1.23 ± 0.25** | **1.28 ± 0.27***** |  |
| V_0_ (rpm) | **209.0 ± 41.6** | **218.4 ± 33.2**** | **215.5 ± 34.6** | **228.1 ± 34.6**** |  |
| P_max_ (W.kg^-1^) | **6.4 ± 1.9** | **7.0 ± 2.0***** | **6.7 ± 1.7** | **7.4 ± 1.7***** |  |
| S_fv_ (N/m.s^-1^) | **-4.2 ± 0.9** | **-4.3 ± 1.2** | **-4.8 ± 1.2** | **-4.7 ± 1.1‡** |  |
