# Supplementary figures and images for: An innovative cardiac rehabilitation based on the power–force–velocity profile to further improve cardiorespiratory capacities in coronary artery disease patients: CITIUS study
Source: Eur Heart J Open. 2025 Apr 22;5(3):oeaf036. doi: 10.1093/ehjopen/oeaf036 (PMC12152306; doi:10.1093/ehjopen/oeaf036)

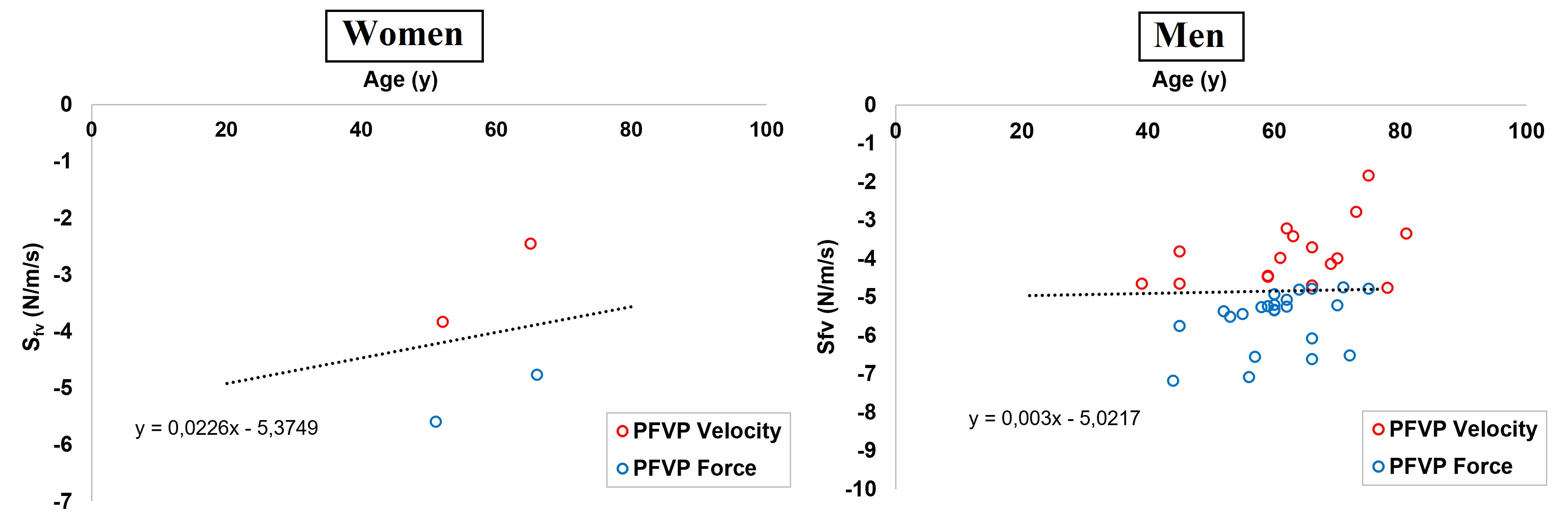

Supplement: oeaf036_Supplementary_Data [file oeaf036_supplementary_data.zip › Figure_S1.tif]
